# Case–control study on the interplay between immunoparalysis and delirium after cardiac surgery

**DOI:** 10.1186/s13019-021-01627-3

**Published:** 2021-08-23

**Authors:** Shokoufeh CheheiliSobbi, Annemieke M. Peters van Ton, Esther M. Wesselink, Marjolein F. Looije, Jelle Gerretsen, Wim J. Morshuis, Arjen J. C. Slooter, Wilson F. Abdo, Peter Pickkers, Mark van den Boogaard

**Affiliations:** 1 Department of Intensive Care Medicine, IP 707, Radboud Institute for Health Sciences, Radboud University Medical Center, P.O. Box 9101, 6500 HB Nijmegen, The Netherlands; 2 Department of Intensive Care Medicine, Radboud Institute for Molecular Life Sciences, Radboud University Medical Center, Nijmegen, The Netherlands; 3 Department of Cardiothoracic Surgery, Radboud University Medical Center, Nijmegen, The Netherlands; 4 Department of Intensive Care Medicine and UMC Utrecht Brain Center, University Medical Center Utrecht, Utrecht University, Utrecht, The Netherlands

**Keywords:** Cardiothoracic surgery, Cytokines, Delirium, Immunity, Intensive care medicine

## Abstract

**Background:**

Delirium occurs frequently following cardiothoracic surgery, and infectious disease is an important risk factor for delirium. Surgery and cardiopulmonary bypass induce suppression of the immune response known as immunoparalysis. We aimed to investigate whether delirious patients had more pronounced immunoparalysis following cardiothoracic surgery than patients without delirium, to explain this delirium-infection association.

**Methods:**

A prospective matched case–control study was performed in two university hospitals. Cytokine production (tumor necrosis factor (TNF)-α, interleukin (IL)-6, IL-8 and IL-10) of ex vivo lipopolysaccharide (LPS)-stimulated whole blood was analyzed in on-pump cardiothoracic surgery patients preoperatively, and at 5 timepoints up to 3 days after cardiothoracic surgery. Delirium was assessed by trained staff using two validated delirium scales and chart review.

**Results:**

A total of 89 patients were screened of whom 14 delirious and 52 non-delirious patients were included. Ex vivo-stimulated production of TNF-α, IL-6, IL-8, and IL-10 was severely suppressed following cardiothoracic surgery compared to pre-surgery. Postoperative release of cytokines in non-delirious patients was attenuated by 84% [IQR: 13–93] for TNF-α, 95% [IQR: 78–98] for IL-6, and 69% [IQR: 55–81] for IL-10. The attenuation in ex vivo-stimulated production of these cytokines was not significantly different in patients with delirium compared to non-delirious patients (*p* > 0.10 for all cytokines).

**Conclusions:**

The post-operative attenuation of ex vivo-stimulated production of pro- and anti-inflammatory cytokines was comparable between patients that developed delirium and those who remained delirium-free after on-pump cardiothoracic surgery. This finding suggests that immunoparalysis is not more common in cardiothoracic surgery patients with delirium compared to those without.

## Introduction

Delirium, characterized by an acute onset of inattention and other cognitive deficits, is a common postoperative complication in cardiothoracic surgery patients with an incidence of 10–20% [1]. Delirium is associated with multiple impaired outcomes, such as increased risk of dementia and prolonged admission with associated costs [2, 3]. Previous studies showed that various pro- and anti-inflammatory mediators may be associated with delirium [4–6], and these may also account for the development of immunoparalysis [7–9]. This suppression of the immune response has also been shown in other states to be associated with severe systemic inflammation, e.g. sepsis, and is associated with an increased susceptibility to secondary infections and morbidity [10]. Interestingly, patients with delirium appear to be prone to develop a secondary infection, but also an infectious disease is a risk factor for delirium [3]. The interplay between immunoparalysis and delirium has not been investigated previously.

Cardiothoracic surgery results in a systemic inflammatory response that could also lead to an innate immune response in the brain, called neuroinflammation [11] that may clinically express as delirium [12]. In addition, several studies have demonstrated that the use of cardiopulmonary bypass (CPB) disturbs the balance between pro- and anti-inflammatory responses, which may induce suppression of the immune responses postoperatively [8, 9, 13, 14].

Given the association between severe inflammation and the development of both delirium and immunoparalysis, we aimed to test the hypothesis that cardiac surgery-induced immunoparalysis is more pronounced in patients that develop delirium compared to those who do not.

## Patients and methods

### Study design and population

This multicenter, prospective, observational case–control study was performed in two University Medical Centers in the Netherlands: Radboud University Medical Center Nijmegen and University Medical Center Utrecht. The study was approved by the medical ethical committees in both centers and the need for informed consent was waived (study number 2012/297 and 12/471).

Eligible patients were aged 50 years or older, scheduled to undergo on-pump coronary artery bypass grafting (CABG) or single heart valve surgery, and delirium-free prior to the operation. Exclusion criteria were combined CABG with valve surgery for reason of group homogeneity, use of blood cardioplegia since this type of cardioplegia is associated with the development of postoperative neurological events [15], a pre-operative diagnosis consistent with immune suppression, inability to screen for delirium, pre-operative infection (determined by positive blood cultures or by administration of antibiotics), and a history of cognitive impairment or psychiatric illness for reason of group homogeneity. Dexamethasone was the only anti-inflammatory medication administrated to all patients during surgery as standard practice in both centers. Cefazolin was used for perioperative antibiotic prophylaxis.

Patients who developed delirium postoperatively were defined as ‘cases’ and patients who did not develop delirium served as ‘non-cases’. The group of cases was matched to non-cases for which propensity matching was used. Propensity scoring parameters were the following pre-operative and postoperative risk factors for delirium [3] gender, age, duration of surgery, extracorporeal circulation (ECC) time, aortic cross clamp (AOX) time, risk of death after cardiac surgery (European System for Cardiac Operative Risk Evaluation (EuroSCORE-II) and severity of illness score (Acute Physiology and Chronic Health Evaluation (APACHE)-II score).

### Delirium diagnosis

Patients were assessed for delirium three times a day by trained nurses and researchers, using validated delirium assessment tools. In the Intensive Care Unit (ICU) the Confusion Assessment Method Intensive Care Unit (CAM-ICU) [16] was used, and on the cardiothoracic surgery ward the Delirium Observation Screening (DOS) scale [17] In order to increase the delirium detection rate, patients’ medical and nursing records were evaluated for delirium signs by the investigators and for delirium treatment. Delirium was defined as having a positive CAM-ICU assessment or a DOS score ≥ 3, use of haloperidol for no other reason than delirium treatment, or when there were typical signs of delirium registered without a positive delirium assessment.

### Data collection and variables

Demographic and clinical data were obtained from electronic patient files. Blood was sampled from the central venous catheter, and if this was not possible, from an indwelling arterial catheter or by vena puncture at six time points: preoperatively, i.e. immediately after the induction of anesthesia, but prior to incision (T_0_), within one hour following post-operative ICU admission (T_1_), 6 h after ICU admission (T_6_), and 24 (T24), 48 (T_48_) and 72 h (T72) after cardiothoracic surgery.

### Ex-vivo cytokine production

Immunoparalysis is characterized by attenuated cytokine production following ex vivo leukocyte stimulation with bacterial lipopolysaccharide (LPS) [7]. Lithium heparin (LH) anti-coagulated blood was obtained for ex vivo stimulation experiments, which were performed immediately after sampling. Leukocyte cytokine production capacity was determined by challenging whole blood from the patients with LPS ex vivo using an in-house developed system with prefilled tubes described in detail elsewhere [18]. Briefly, 0.5 mL of blood was added to tubes prefilled with 2 mL culture medium as negative control or 2 mL culture medium supplemented with *Escherichia coli* LPS (serotype O55:B5 (Sigma Aldrich, St Louis, MO, USA)), resulting in a final LPS concentration of 10 ng/mL. Cultures were incubated at 37 °C for 24 h, centrifuged, and supernatants were stored at − 80 °C until analysis. Concentrations of IL-6, IL-8, IL-10 and TNF-α, were determined batchwise by ELISA according to the manufacturer’s instructions (R&D systems, Minneapolis, MN, USA). Cytokine concentrations were normalized to monocyte counts since these cells are the main cytokine-producing cells in whole blood stimulation assays [19].

### Statistical analysis

Demographic and patient characteristics were reported as means with standard deviation (SD), medians with interquartile range [IQR], or frequencies, depending on data distribution or occurrence. Accordingly, the Student’s t-test, Mann–Whitney U test, or Fischer’s exact test were used for group comparisons. Propensity scores were calculated using logistic regression analysis with ‘delirium’ or ‘no delirium’ as dependent variable and the delirium risk factors; age, APACHE-II score, AOX-time and EuroSCORE-II, as independent variables. Linear mixed effects models were used to study associations between the trajectory of log-transformed ex vivo cytokine concentrations and the occurrence of postoperative delirium. The time of sampling and the occurrence of delirium (yes/no) were analyzed as fixed effects, while subject ID was entered as random effect. Statistical significance was defined as a *p* value of < 0.05. Statistical analysis was performed using R, version 3.6.2 (http://www.R-project.org) software packages, and IBM SPSS Statistics 20 (IBM, NY, USA). Figures were created in GraphPad Prism 5.0 (Graphpad Software, San Diego, CA, USA).

## Results

### Patient characteristics

A total of 89 cardiothoracic surgery patients were screened of whom 18 (20%) developed delirium postoperatively. One non-delirious patient was excluded due to a postoperative cerebral infarction, twelve non-delirious and four delirious patients were excluded because of absent cytokine data. Subsequently 14 cases were matched with 52 non-delirious cases. Nine patients developed delirium within 24 h and five patients developed delirium within 72 h after surgery. No significant differences were found in demographic characteristics between delirious and non-delirious patients (Table 1). None of the participants were discharged on antipsychotic therapy.

**Table 1:** Demographic variables of delirious and non-delirious patients

	Non-delirious patients (n = 52)	Delirious patients (n = 14)	*p*-value
Age (years), mean (SD)	65 (8.2)	68 (6.9)	0.196^a^
Sex, male, n (%)	37 (71)	9 (64)	0.745^b^
BMI, kg/m^2^, mean (SD)	28 (4.7)	28 (4.3)	0.636^a^
Type of surgery			0.536^b^
CABG, n (%)	35 (67)	8 (57)	
Aortic valve, n (%)	17 (33)	6 (43)	
Duration of surgery, minutes, mean (SD)	203 (53)	196 (65)	0.700^a^
Perfusion time, minutes, median [IQR]	90 [72–107]	82 [69–117]	0.790^c^
AOX time, median [IQR]	63 [52–78]	62 [48–75]	0.505^c^
APACHE-II score, median [IQR]	17 [14–29]	19 [16–42]	0.243^c^
EuroSCORE-II, mean (SD)	3.4 (2.3)	4.3 (2.0)	0.209^a^
Preoperative plasma creatinine level, μmol/L, median [IQR]	84 [75–96]	85 [70–103]	0.947^c^

AOX, aortic cross-clamping; APACHE-II, Acute Physiology and Chronic Health Evaluation II score; BMI, body mass index; CABG, coronary artery bypass grafting surgery; EuroSCORE-II, European System for Cardiac Operative Risk Evaluation-II; IQR, interquartile range; SD, standard deviation

^a^Unpaired t-test; ^b^Fisher’s exact test; ^c^Mann–Whitney U test

### Ex vivo *cytokine production*

**Fig. 1:**
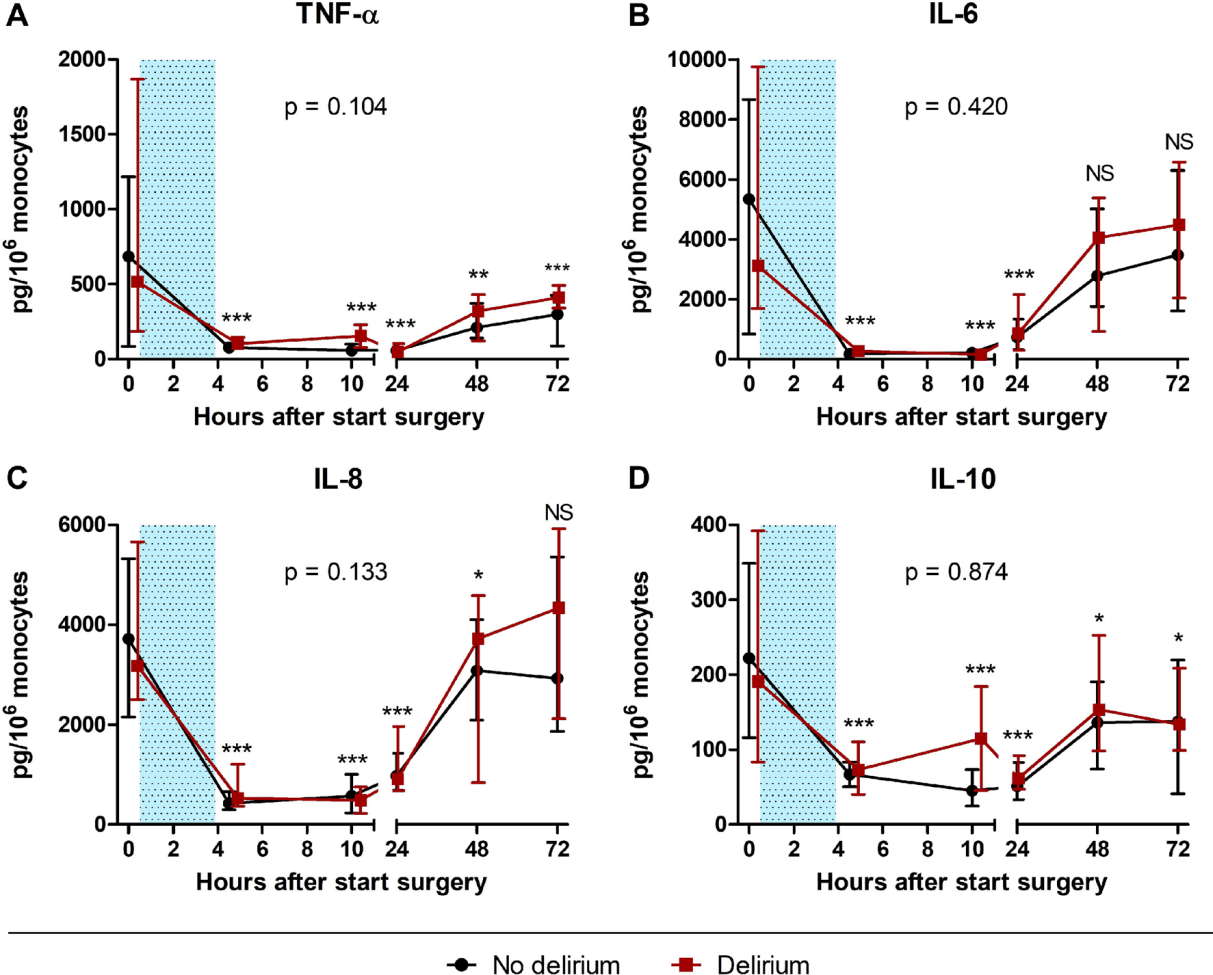
Cytokine producing capacity in delirious and non-delirious patients, after whole blood ex vivo-stimulation with bacterial lipopolysaccharide. **A** Tumor necrosis factor-α (TNF-α). **B** Interleukin-6 (IL-6)*.* **C** IL-8*.* **D** IL-10*.* Data are expressed as median concentration per 10^6^ monocytes [interquartile range]. The blue dotted rectangles illustrate the duration of surgery. *p < 0.05; **p < 0.01; ***p < 0.001, NS: no significant change from baseline, according to linear mixed model analysis (LMM) with time of sampling and delirium occurrence as fixed variables, and subject ID as random effect. The *p*-values above each graph indicate no significant differences in the trajectories of ex vivo cytokine production between patients with and without postoperative delirium based on the LMM analysis, described above

Both groups showed a swift, profound and statistically significant attenuation of ex vivo-stimulated cytokine (TNF-α, IL-6, IL-8, and IL-10) production directly at ICU admission after surgery (Fig. 1). For all cytokines, the recovery of cytokine producing capacity started from 24 h onwards. However, TNF-α and IL-10 productions were still significantly suppressed 72 h after surgery compared to the first preoperative baseline measurement (− 44% [95% CI − 60 to − 21%], *p* < 0.001, and − 33% [95% CI − 51 to − 9%], *p* = 0.01, respectively).

Ex vivo-stimulated cytokine production pre- and postoperatively were not significantly different between delirious and non-delirious patients (difference between groups: TNF-α (*p* = 0.10), IL-6 (*p* = 0.42), IL-8.

(*p* = 0.13), and IL-10 (*p* = 0.87), Fig. 1).

## Discussion

This study suggests that the extent of postoperative immunoparalysis is similar in patients that develop delirium, compared to those who do not. This indicates that immunoparalysis does not play an important role in the pathogenesis of delirium and presumably not in the development of secondary infections that are more frequently observed in patients suffering from delirium. In addition, our data confirms previous studies [14, 20–23] that the ex vivo-stimulated production of both pro-inflammatory (IL-6, IL-8 and TNF-α) and anti-inflammatory (IL-10) cytokines are severely suppressed following on-pump cardiothoracic surgery.

To our knowledge, it has not been studied previously to what extent ex vivo-stimulated cytokine production is attenuated in patients with and without delirium following cardiac surgery. Elevated plasma concentrations of TNF-α, IL-6 and IL-8 were reported to be associated with delirium [4–6, 24, 25]. In our study we did not analyze the in vivo plasma levels of pro- and anti-inflammatory cytokines since our aim was to determine whether or not immunoparalysis was more pronounced in patients with delirium after cardiothoracic surgery. Additionally, plasma concentrations of cytokines are not a sensitive hallmark of immunoparalysis [26].

This study has several limitations. First, although delirium was not diagnosed by an delirium expert such as a psychiatrist, neurologist or geriatrician, we used validated and frequently used delirium assessment tools for this purpose [16, 17], that were applied by trained (ICU) nurses and researchers. This was complemented by a detailed assessment of the medical and nursing files for notes on delirium and treatment with haloperidol in order not to miss any patients with delirium. Second is the relative small number of patients. However, with our standardized and robust methodology of ex vivo immunostimulation, combined with our matched case–control design, we were able to find a homogenous immune response with small margin of error between patients with and without delirium. Based on our data, we do not expect that a larger number of patients would likely change our results. Third, in the current study all patients were treated with dexamethasone per-operatively, while dexamethasone inhibits LPS-stimulated production of TNF-α, IL-6, IL-8 and IL-10 [27–29]. However, previous research demonstrated that immunoparalysis occurs after cardiothoracic surgery irrespective of corticosteroids use [14, 20–23]. Finally, the incidence of secondary infections after cardiac surgery is low [30, 31], and the power of this cohort was insufficient to analyze associations between delirium, secondary infections and immunoparalysis. This renders us unable to draw conclusions on whether or not immunoparalysis is involved in the pathogenesis of secondary infections that are more frequently observed in patients suffering from delirium. Alternatively, acute malnutrition, dehydration and aspiration pneumonia are common in delirious patients and may explain the increased susceptibility to secondary infections in delirium [32].

## Conclusion

The time-course of ex vivo-stimulated production of pro- and anti-inflammatory cytokines was comparable between delirious and non-delirious patients in on-pump post cardiothoracic surgery patients. This finding indicates that immunoparalysis is not more pronounced in cardiothoracic surgery patients with delirium than in those without.

## Data Availability

The datasets analyzed during the current study are available from the corresponding author on reasonable request.

## Abbreviations

AOX: Aortic cross clamp
APACHE: Acute Physiology and Chronic Health Evaluation
BMI: Body mass index
CABG: Coronary artery bypass grafting
CAM-ICU: Confusion Assessment Method Intensive Care Unit
CPB: Cardiopulmonary bypass
DOS: Delirium Observation Screening
ECC: Extracorporeal circulation
EuroSCORE-II score: European System for Cardiac Operative Risk Evaluation
ICU: Intensive Care Unit
IL: Interleukin
IQR: Interquartile range
LPS: Lipopolysaccharide
LH: Lithium heparin
N/A: Not applicable
SD: Standard deviation
TNF: Tumor necrosis factor
